# Ageism Predicts Prioritizing COVID-19 Vaccines for Older Adults and LTC Residents

**DOI:** 10.1093/geroni/igab046.2301

**Published:** 2021-12-17

**Authors:** Carly Pullen, Jenessa Steele, Julie Hicks Patrick

**Affiliations:** 1 West Virginia University , Morgantown, West Virginia, United States; 2 Radford University, Radford, Virginia, United States; 3 West Virginia University, Morgantown, West Virginia, United States

## Abstract

Ageism and ageistic stereotypes regarding older adults have become widespread and influence many policies and practices. Benevolent ageism includes attitudes or behaviors that appear overtly positive but are actually patronizing (Cary et al., 2017). Hostile ageism, usually expressed through negative stereotypes, highlights older adults' poor physical and mental functioning (Cary et al., 2017). The aim of the current study was to examine the role of benevolent and hostile ageism on perceptions of vaccination priority during the COVID-19 pandemic. As part of a larger longitudinal study on perceptions of aging, measures of benevolent and hostile ageism were collected in January 2020, before the pandemic began in the US. In March 2021, the same participants were asked to identify top vaccine priorities by ranking groups (e.g., adults 65 and older, school teachers, etc.). Participants who put older adults in the lowest priority group expressed more benevolent ageism before the pandemic than those who put older adults in the top priority group, F(2, 88) = 3.93, p < .05. Participants who put older adults in the lowest priority group expressed more hostile ageism prior to the pandemic, compared to those who put older adults in the top priority group, F(2, 88) = -3.34, p < .05. Similar to Vale and colleague (2020) findings, our results suggest that ageism influences people's ideas about triage for the vaccine. Notably, neither form of ageism related to prioritization for other high-risk groups, including members of racial/ethnic minority groups or health care workers.

